# A Fetal Electrocardiogram Signal Extraction Algorithm Based on Fast One-Unit Independent Component Analysis with Reference

**DOI:** 10.1155/2016/5127978

**Published:** 2016-09-15

**Authors:** Yanfei Jia, Xiaodong Yang

**Affiliations:** ^1^College of Information and Communication Engineering, Harbin Engineering University, Heilongjiang 150001, China; ^2^College of Electrical and Information Engineering, Beihua University, Jilin 132012, China; ^3^Collaborative Research Center, Meisei University, Tokyo 1918506, Japan

## Abstract

Fetal electrocardiogram (FECG) extraction is very important procedure for fetal health assessment. In this article, we propose a fast one-unit independent component analysis with reference (ICA-R) that is suitable to extract the FECG. Most previous ICA-R algorithms only focused on how to optimize the cost function of the ICA-R and payed little attention to the improvement of cost function. They did not fully take advantage of the prior information about the desired signal to improve the ICA-R. In this paper, we first use the kurtosis information of the desired FECG signal to simplify the non-Gaussian measurement function and then construct a new cost function by directly using a nonquadratic function of the extracted signal to measure its non-Gaussianity. The new cost function does not involve the computation of the difference between the function of the Gaussian random vector and that of the extracted signal, which is time consuming. Centering and whitening are also used to preprocess the observed signal to further reduce the computation complexity. While the proposed method has the same error performance as other improved one-unit ICA-R methods, it actually has lower computation complexity than those other methods. Simulations are performed separately on artificial and real-world electrocardiogram signals.

## 1. Introduction

The fetal electrocardiogram (FECG) contains much important information about the health and possible diseases of the fetus, which reflects the complete view of the heart activities. Additionally, it is more sensitive than color Doppler ultrasound in the case of fetal acidosis and anoxia. With the analysis of the FECG, the doctor can discover fetal hypoxemia, umbilical abnormality, and other fetal abnormality situations timely and take early effective actions to ensure the health of the fetus. However, the FECG signal is considerably weaker than the maternal electrocardiogram (MECG) and is often embedded in the noise, MECG, baseline wandering, power line interference, and so forth. Accordingly, it is quite difficult to extract the FECG signal and further reduce the diagnostic accuracy.

A number of methods have been reported for extracting the FECG signal, like filtering method [1], singular value decomposition [2], wavelet transform [3], independent component analysis (ICA) [4], and others [5]. Every method has its advantages and disadvantages [5, 6]. In this paper, we only consider the method based on ICA. The FECG extraction based on the ICA methods considers the extracting FECG signal as a blind source separation problem. They consider the unknown FECG signal, MECG, and other interference signals as source signals and the measured signals from the maternal abdomen and chest as mixed signals (measured signals). It actually can separate all source signals, including the FECG signal and MECG signal. Compared with the other methods, it has a simple structure and has been proved to be efficient in extracting FECG in many studies [4–9]. However, for extracting the FECG, the ICA method must first separate all the source signals from the measured signals and then artificially selects the FECG signal. When the number of measured signals is very large, the extraction of the FECG signal will consume plenty of time.

To overcome this problem, the independent component analysis with reference (ICA-R) is applied to extract the FECG [10–14], which separates the desired FECG signal by using some prior information about the desired FECG without separating all the source signals. Therefore, this FECG extraction method based on ICA-R is more efficient than those based on ICA. ICA-R was proposed and implemented by Lu and Rajapakse [15–17] by incorporating the prior information of the desired signal into the contrast function of the ICA [18]. To improve the ICA-R performance, some authors proposed improved ICA-R methods. For example, to reduce the computational complexity of ICA-R, Lin et al. [11] proposed a fast one-unit ICA-R by using prewhitening to deal with the observed signal. Compared with the original ICA-R, this method has shorter running time and the same error. Also, Huang and Mi [19] studied the gradient of inequality constraint function and corrected the gradient. This method has a higher success rate for extracting the desired signal. In addition, Li et al. [14] first divided the cost function of ICA-R into two parts that are the negentropy function and the closeness measure function and then directly used the first-order derivative of the negentropy contrast function to update the separating vector. This method has faster convergence rate and higher success rate to extract the desired signal than the previously reported method [19]. Moreover, Sun and Shang [20] studied the stability of ICA-R and proposed an initialization method of separating the matrix. This method has a higher accuracy and stability than the original ICA-R. Kavuri et al. [21] proposed a one-unit ICA-R by using evolutionary algorithm to optimize the cost function to find a global optimal solution. Although this method has a smaller error than the original ICA-R, it does consume a considerable amount of time. Additionally, Zhang et al. [22] proposed the ICA-R based on kurtosis and analyzed how to choose the reference signal. Wang [23] used the method that has been used in the fast ICA to propose a fixed-point ICA method. Rodriguez et al. [24] proposed a multiunit ICA-R method based on nonorthogonal decoupling of separated matrix. This method cannot be extended to one-unit ICA-R. Mi [25] proposed a strategy to detect future misconvergence to improve the probability of extracting the desired signal. Chen et al. [26] proposed an ICA-R that can be used in single channel by discrete wavelet transform.

The one-unit ICA-R is a special case of the multiunit ICA-R. It only separates one desired source signal every time. The only difference between the one-unit ICA-R and the multiunit ICA-R is that the former only extracts one desired signal, and the latter is based on the former and uses the orthogonalization method to extract multiple desired signals. If we use the deflationary orthogonalization method or symmetric orthogonalization method, which are widely used in ICA [18] to extract the signal in the one-unit ICA-R, the one-unit ICA-R can be easily extended to multiunit ICA-R. In the FECG extraction, we only want to extract the FECG with just one signal; thus, the one-unit ICA-R is enough to extract FECG.

Although the one-unit ICA-R and multiunit ICA-R have a little difference, they share the same cost function. Additionally, they both use the same non-Gaussian measurement function that is used in the classical ICA algorithm to measure the non-Gaussian component of the extracted signal. The negentropy approximation is needed to compute the difference between the function of the extracted signal and the function of a Gaussian vector that has the same mean and variance as the extracted signal. This will consume plenty of time. The classical ICA algorithm has no information about the source signals, except that the non-Gaussian measurements and statistics are independent. However, the ICA-R has prior information about the desired signal, but the previous methods did not use such information to reduce the computational complexity of the negentropy approximation. The aim of this paper is to reduce the computational complexity for the one-unit ICA-R by taking advantage of the prior information of the desired ECG to simplify the negentropy approximation.

This paper is organized as follows.  Section 2 reviews the previously reported algorithms including the traditional ICA and one-unit ICA-R.  Section 3 analyzes in detail the non-Gaussian measurement function that is used in the one-unit ICA-R cost function. In Section 4, we simplify the non-Gaussian measurement function based on the analysis in Section 3 and, then, derive an improved one-unit ICA-R with lower computation complexity. In Section 5, we test Mi's method [25] and our proposed method on artificial ECG data and real-world ECG data to compare the performance of each method. Some conclusions are included in Section 6.

## 2. Previous Algorithms

### 2.1. Traditional ICA

The traditional ICA is a signal processing method for estimating the source signals from observed signals that are a mixture of source signals. The model of linear ICA is as follows:

(1) $$\begin{array}{c} x\left(\;t\;\right)=As\left(\;t\;\right), \end{array}$$

where **x**(*t*) = [*x*_1_(*t*), *x*_2_(*t*),…, *x*_*n*_(*t*)]^*T*^ is the observed signal, **A** is the mixing matrix with the size (*n* × *m*), and **s**(*t*) = [*s*_1_(*t*), *s*_2_(*t*),…, *s*_*m*_(*t*)]^*T*^ is the source signal. The aim of the ICA is to estimate the source signal **s**(*t*) from the observed signal **x**(*t*) by computing the separating matrix **W** = [**w**_1_, **w**_2_,…, **w**_*m*_]^*T*^ with size (*m* × *n*). The estimated source signal can be expressed as follows:

(2) $$\begin{array}{c} y\left(\;t\;\right)=Wx\left(\;t\;\right), \end{array}$$

where **y**(*t*) = [*y*_1_(*t*), *y*_2_(*t*),…, *y*_*m*_(*t*)]^*T*^ is the estimated source signal, which is the same as **s**(*t*) under ideal condition. However, under real condition, the shape of the waveforms of **y**(*t*) is close to **s**(*t*), except for the amplitude and sequence of the waveforms. The source signals **s**(*t*) are considered as non-Gaussian and mutually statistically independent. Accordingly, it has strong non-Gaussian properties. The mixing signals **x**(*t*) have strong Gaussian characteristics according to the central limit theorem. When the estimated source signal is close to the source signal **s**(*t*), it will have strong non-Gaussian properties. Thus, the ICA uses a non-Gaussian measurement function of the estimated source signals as a cost function [18]:

(3) $$\begin{array}{c} J\left(\;y\;\right)\propto \rho {\left[\;E\left\{\;F\left(\;y\;\right)\;\right\}-E\left\{\;F\left(\;v\;\right)\;\right\}\;\right]}^{2}, \end{array}$$

where *ρ* is a positive constant, *y* is the estimated signal (extracted signal), *F* is the nonquadratic function, and *v* is a Gaussian variable with the same mean and variance as *y*. By maximizing the cost function, we can obtain the separating matrix and recover the source signal.

### 2.2. One-Unit ICA-R

The ICA can separate all the source signals from the observed signals. However, if we only want to extract some or one of the desired source signals, we must use ICA to separate all source signals and further select the desired source signal from the estimated signals. When the dimension of observed signals is very large, especially for some biomedical signals, it will take a long period of time to extract the desired signal. The one-unit ICA-R was proposed by Lu and Rajapakse [15] to reduce computational time and avoid additional operations to select the desired source signal from the estimated signals.

The one-unit ICA-R method combines prior information of the desired source signal into the ICA cost function (3) and constructs a new cost function. The cost function for the one-unit ICA-R is as follows [15]:

(4) max⁡ Jy≈ρEFy−EFv2s.t. gy≤0 hy=Ey2−1=0,

where *y* = **w** ^*T*^ **x**, **w** is the separating vector, **x** is the observed signals, *y* is the extracted signal, *ρ* is a positive constant, and *v* is a Gaussian vector with the same mean and variance as *y*. The inequality constraint term is *g*(*y*) = *ε*(*y*, *r*) − *ξ*, where *r* is the reference signal constructed by the prior information of desired signal, *ε* is used to measure the closeness between the estimated signal *y* and the reference signal *r*, and  *ξ* is a threshold parameter to control the closeness level. A common and simple measure of closeness is the mean squared error (MSE) *ε*(*y*, *r*) = *E*{(*y* − *r*)^2^}. *h*(*y*) is used to restrict the estimated signal *y* to unit variance.

The above inequality constraints can be transformed into equality constraints by introducing the slack factor *z*. Thus, the cost function of the one-unit ICA with reference to equality constraint can be expressed as follows:

(5) max⁡ Jy≈ρEFy−EFv2s.t. gy+z2=0 hy=Ey2−1=0.

The augmented Lagrange multiplier method is used to optimize the above cost function with equality constraint and derive the augmented Lagrange function that can be expressed as follows:

(6) L=Jy−12γmax⁡γgy+μ,02+μ22γ−λhy−0.5γhy2,

where *μ* and *λ* are the Lagrange multipliers for the constrains *g*(*y*) and *h*(*y*), respectively, and *γ* is a scalar penalty parameter. A Newton-like learning algorithm is applied to optimize the above equation and get the updated separating vector. The updated formula is as follows [15]:

(7) $$\begin{array}{c} w_{k+1}=w_{k}-\eta R^{-1}\frac{l}{\delta }, \end{array}$$

where *k* is the current iterative number, *η* is the fixed learning rate, **R** is the covariance matrix of the observed signals, and *l* and *δ* are the first and second derivatives of *l*, respectively, and can be expressed as

(8) l=ρ^Exfy−0.5μExg′y−λExyδ=ρ^Ef′y−0.5μEg′′y−λ,

where $\hat{\rho }=2\rho [E\{F(y)\}-E\{F(v)\}]$, *f*(*y*) and *f*′′(*y*) are the first and the second derivatives of *F*(*y*) with respect to *y*, respectively, and *g*′(*y*) and *g*′′(*y*) are the first and the second derivatives of *g*(*y*) with respect to *y*, respectively.

The optimum multipliers *μ* and *λ* are updated by the following equations:

(9) μk+1=max⁡0,μk+γgy

(10) λk+1=λk+γhy.

The same author Lu and Rajapakse proposed [17] another cost function for the one-unit ICA-R, as follows:

(11) min⁡ −Jy≈−ρEFy−EFv2s.t. gy≤0 hy=Ey2−12=0.

The learning rule of the separating vector is

(12) $$\begin{array}{c} w_{k+1}=w_{k}-\eta R^{-1}\frac{l}{\delta }, \end{array}$$

where

(13) l=ρ^Exfy−μExg′y−4λEy2−1Exyδ=ρ^Ef′y−μEg′′y−8λ.

The symbols of the parameters in (12) have the same meaning as those in (7). Many studies about the ICA-R method are based on the cost function (11) [19, 20, 25].

## 3. Analysis of the Non-Gaussian Measurement Function in the One-Unit ICA-R Method

The non-Gaussian measurement function (5) that is also the cost function of the fast ICA can also be expressed as

(14) $$\begin{array}{c} J\left(\;y\;\right)=\rho \left|\;E\left\{\;F\left(\;y\;\right)\;\right\}-E\left\{\;F\left(\;v\;\right)\;\right\}\;\right|, \end{array}$$

where |(·)| is the absolute value of (·). Maximizing (14) equals maximizing (3). In ICA-R, *v* is a Gaussian variable having zero mean and unit variance [17]. It actually has nothing to do with the separating vector **w** and can be seen as a constant for **w**. Accordingly, maximizing (14) equals maximizing or minimizing *E*{*F*(*y*)}. We denote it by *H*(*y*); that is,

(15) $$\begin{array}{c} H\left(\;y\;\right)=E\left\{\;F\left(\;y\;\right)\;\right\}, \end{array}$$

where *y* = **w** ^*T*^ **x** = **w** ^*T*^ **A** **s** = **b** ^*T*^ **s**, **s** is the source signals, **A** is the mixing matrix, and **w** is the separating vector. In the one-unit ICA-R, we only extract one desired signal. We consider that the successfully extracted signal *y* equals *s*_*i*_; hence, the stability point of **b** is **b** ^*∗*^ = (0_0_,…, 0_*i*−1_, 1, 0_*i*+1_,…)^*T*^.

At the stability point, the first-order derivative of *H*(**b** ^*T*^ **s**) with respect to **b** ^*∗*^ is

(16) ∇Hb∗TsEsfb∗Ts=Esfsi=01,…,0i−1,1,0i+1,…TEsifsi.

The *E*{*f*(*s*_*i*_)*s*_*j*_}_*i*≠*j*_ = 0, which means that the source signals with zero are mutually statistically independent, is used in (16). It is a fundamental assumption in ICA-R [17] and its second derivative is as follows:

(17) ∇2Hb∗Ts=EssTf′b∗Ts=diag⁡Ef′si,…,Esi2f′si,…,Ef′si.

Adding a small fluctuation **σ** = (*σ*_1_,…, *σ*_*i*−1_, *σ*_*i*_, *σ*_*i*+1_,…) into **b** ^*∗*^, we obtain

(18) $$\begin{array}{c} b_{new}=b^{*}+\sigma =\left(\;\sigma_{1},\ldots ,\sigma_{i-1},\sigma_{i}+1,\sigma_{i+1},\ldots \;\right). \end{array}$$

Taylor's series expansion of *H*(**b**_new_^*T*^ **s**) at the point **b** ^*∗*^ is

(19) Hb∗+σTs=Hb∗Ts+σ∇Hb∗Ts+12σ∇2Hb∗TsσT+oσ2=Hb∗+σiEsifsi+12Esi2f′siσi2+Ef′si∑n≠i σn2+oσ2.

In the one-unit ICA-R, the extracted signal *y* is considered to have unit variance. When the desired signal has unit variance, then ‖**b** ^*∗*^ + **σ**‖ = **I**. That is, *σ*_1_^2^ + *σ*_2_^2^ + ⋯+(*σ*_*i*_ + 1)^2^ + ⋯ = 1. Thus, we obtain $(\sigma_{i}+1)=\sqrt{1-\sigma_{1}^{2}\cdots -\sigma_{i-1}^{2}-\sigma_{i+1}^{2}\cdots }$  and its Taylor's series at point zero is

(20) $$\begin{array}{c} \sigma_{i}+1=1-\frac{1}{2}\underset{n\neq i}{\sum }\sigma_{n}^{2}+o\left(\;\underset{n\neq i}{\sum }\sigma_{n}^{2}\;\right). \end{array}$$

Substituting (20) in (19), we get

(21) Hb∗+σTs=Hb∗Ts−12Esifsi∑n≠iσn2+12Esi2f′siσi2+Ef′si∑n≠iσn2+oσ2=Hb∗Ts+12Ef′si−Esifsi∑n≠iσn2+oσ2≈Hb∗Ts+12Ef′si−Esifsi∑n≠iσn2.

As the components of **σ** are very small, we can neglect its higher order term in (20) and (21). From the last item in (21), we can obtain that when *E*{*f*′(*s*_*i*_)} − *E*{*s*_*i*_ *f*(*s*_*i*_)} > 0 then *H*(**b** ^*∗*^) is the minimum, and vice versa is the maximum.

## 4. Improve One-Unit ICA-R Method

As the previous one-unit ICA-R methods involve the computation of the $\hat{\rho }=2\rho [E\{F(y)\}-E\{F(v)\}]$ in every iteration, it is a time consuming procedure. If the computation of [*E*{*F*(*y*)} − *E*{*F*(*v*)}] is avoided in $\hat{\rho }$, the implementation of the ICA algorithm and ICA-R algorithm can be further speeded up. We wish to incorporate the prior information of the desired signal into non-Gaussian measurement function *J*(*y*) to eliminate *E*{*F*(*v*)} to avoid further computing [*E*{*F*(*y*)} − *E*{*F*(*v*)}]. *J*(*y*) is a part of the cost function of the one-unit ICA-R. As a result, this will reduce computation complexity of the one-unit ICA-R.

### 4.1. Simplification of the Non-Gaussian Measurement Function

Since the nonlinear function *F* used in one-unit ICA-R is a positive even function, the terms *E*{*F*(*v*)} and *E*{*F*(*y*)} are both positive in (14), and *v* has nothing to do with the separating vector and thus can be seen as a constant. Accordingly, when *E*{*F*(*y*)} has a maximum value, this equals [*E*{*F*(*y*)} − *E*{*F*(*v*)}], which has a maximum value. On the contrary, when *E*{*F*(*y*)} has a minimum value, this equals −[*E*{*F*(*y*)} − *E*{*F*(*v*)}], which has maximum value.

Based on the analysis in Section 3, when *E*{*f*′(*s*_*i*_)} − *E*{*s*_*i*_ *f*(*s*_*i*_)} < 0, *E*{*F*(*y*)} has maximum value. Therefore, when *E*{*f*′(*s*_*i*_)} − *E*{*s*_*i*_ *f*(*s*_*i*_)} < 0, we can determine that [*E*{*F*(*y*)} − *E*{*F*(*v*)}] has a maximum value based on the analysis described in the first paragraph of this subsection. Based on the above analysis, when *E*{*f*′(*s*_*i*_)} − *E*{*s*_*i*_ *f*(*s*_*i*_)} < 0, then (14) can be simplified as

(22) $$\begin{array}{c} J\left(\;y\;\right)=\rho \left[\;E\left\{\;F\left(\;y\;\right)\;\right\}-E\left\{\;F\left(\;v\;\right)\;\right\}\;\right]. \end{array}$$

As *v* has nothing to do with the separating vector, we can further simplify the non-Gaussian measurement function (22) as

(23) $$\begin{array}{c} J\left(\;y\;\right)=\rho E\left\{\;F\left(\;y\;\right)\;\right\}. \end{array}$$

On the other hand, when the *E*{*f*′(*s*_*i*_)} − *E*{*s*_*i*_ *f*(*s*_*i*_)} > 0, the non-Gaussian measurement function can be simplified as

(24) $$\begin{array}{c} J\left(\;y\;\right)=-\rho E\left\{\;F\left(\;y\;\right)\;\right\}. \end{array}$$

Therefore, the negentropy approximation in (23) and (24) can be expressed as

(25) $$\begin{array}{c} J\left(\;y\;\right)=\rho \;sign\left(\;c\;\right)E\left\{\;F\left(\;y\;\right)\;\right\}, \end{array}$$

where *c* = *E*{*s*_*i*_ *f*(*s*_*i*_)} − *E*{*f*′(*s*_*i*_)}.

(26) $$\begin{array}{c} sign\left(\;c\;\right)=\left\{\begin{array}{cc} 1 & c>0\\ -1 & c<0. \end{array}\right. \end{array}$$

In [16], the following function *F*(*y*) was proposed for extracting the super-Gaussian and sub-Gaussian signal, respectively.

(27) Fsupy=1alogcosh⁡ay−a2y2

(28) Fsuby=b4y4,

where *a*, *b* ∈ *R* ^+^. In [25], Mi has proved that (27) should be corrected as

(29) $$\begin{array}{c} F_{sup}\left(\;y\;\right)=\frac{1}{a}log\cosh\left(\;ay\;\right). \end{array}$$

Accordingly, in this paper, we use (29) instead of (27). For simplicity, we set *a* = 1 in (29). The first and second derivatives are tanh⁡(*y*) and 1 − tanh^2^⁡(*y*), respectively.

If we use (29) as a nonlinear function, *c* in (25) can be expressed as follows:

(30) cEsifsi−Ef′si=Esitanh⁡si−E1−tanh2⁡si=Esi2−si43+osi6−1+Esi2−2si43+osi6.

In (30), we used Taylor's series expansion of tanh⁡(*s*_*i*_), which is described as

(31) $$\begin{array}{c} \tanh\left(\;s_{i}\;\right)=s_{i}-\frac{s_{i}^{3}}{3}+o\left(\;s_{i}^{5}\;\right). \end{array}$$

Neglecting the higher order term in (30) and considering that the desired signal has unit variance, which is a very common hypothesis in ICA and ICA-R, we can obtain the following:

(32) cEsi2−si43−1+Esi2−2si43=E2si2−1−Esi4=1−Esi4.

According to the kurtosis definition of a super-Gaussian signal, when *s*_*i*_ is a super-Gaussian signal with unit variance, *E*{*s*_*i*_^4^} > 3. Then *c* < 0 in (32). This means that if the desired signal is super-Gaussian, we can directly simplify (25) as

(33) $$\begin{array}{c} J\left(\;y\;\right)=-\rho E\left\{\;F\left(\;y\;\right)\;\right\}. \end{array}$$

### 4.2. Improved One-Unit ICA-R for Extracting FECG

The one-unit ICA-R is used to extract one desired signal. The prior information that the desired signal is a super-Gaussian or a sub-Gaussian signal is easily obtained. The FECG and MECG are both super-Gaussian signals in general. Therefore, in the extraction of FECG, we can directly use (33) instead of (14) to obtain the non-Gaussian measurement of an extracted signal. Centering and whitening can reduce the computation complexity of one-unit ICA-R [11]. Thus, we use centering and whitening to process the observed signals. After preprocessing, the new cost function of the one-unit ICA-R for extracting FECG can be expressed as

(34) min⁡ −Jy≈ρEFys.t. gy≤0,

where *y* = **w** ^*T*^ **x** is the extracted signal, **x** is the centered and whitened signal, and **w** is the separating vector. *ρ* in (34) is different from $\hat{\rho }$ in (12). *ρ* is just a positive constant. Correspondingly, the augmented Lagrangian function *L*(*w*, *μ*) for the problem in (34) is expressed as follows:

(35) $$\begin{array}{c} L\left(\;w,\mu \;\right)=J\left(\;y\;\right)-\frac{1}{2\gamma }\left[\;{max}^{2}\;\left\{\;rg+\mu ,0\;\right\}-\mu^{2}\;\right]. \end{array}$$

To find the maximum of *L*(*w*, *μ*), Newton's method is used to optimize *L*(*w*, *μ*). The learning rule of the separating vector *w* is given by the following equation:

(36) $$\begin{array}{c} w_{k+1}=w_{k}-\eta \frac{l}{\delta }, \end{array}$$

where *η* is the learning rate:

(37) l=ρExfy+μExg′y,δ=ρEf′y+μEg′′y,gy=εy,r−ξ.

The main difference between the improved algorithm in (36) and the original algorithm in (12) is that *ρ* in (36) is just a positive constant and $\hat{\rho }$ in (12) is 2*ρ*(*E*{*F*(*y*)} − *E*{*F*(*v*)}). In the original algorithm, we have to additionally compute (*E*{*F*(*y*)} − *E*{*F*(*v*)}) in every iteration, which considerably consumes much more time compared with the improved algorithm.

The one-unit ICA-R uses Newton's method to optimize cost function. Newton's method is sensitive for the initial value. The initial separating vector directly affects the convergence of Newton's method. However, the original algorithm randomly initializes the separating vector. To avoid random initialization, inspired by previous a report [20], we use the following expression to initialize the separating vector:

(38) $$\begin{array}{c} w_{0}={\left(\;rx^{-1}\;\right)}^{T}, \end{array}$$

where **r** is the whitened reference signal, **x** is the whitened signal, **x** ^−1^ is the inversion of **x**, and **w**_0_ is the initialization separating vector. The closer to whitened desired signal **r** is, the closer to the perfect separating vector **w**_0_ will be. This can speed up the algorithm convergence rate and improve the probability of global convergence compared with random initialization.

Therefore, the proposed one-unit ICA-R algorithm for extracting FECG can be described as follows:

(1) Center the observed signals.
(2) Whiten the centered signal.
(3) Choose a proper scalar penalty parameter *γ*, convergence threshold *ς*, parameter *ρ*, and the leaning rate *η*; generally let *ρ* = 1, *η* = 1.
(4) Use (38) to initialize separating vector **w**_0_.
(5) Normalize **w**_0_ ← **w**_0_/‖**w**_0_‖.
(6) Choose an initial value for *μ*.
(7) Update the Lagrange multiplier *μ* by (9).
(8) Update the separating vector **w** by (36).
(9) Normalize the separating vector *w* by
  (39) $$\begin{array}{c} w_{k+1}⟵\frac{w_{k+1}}{\left\|w_{k+1}\right\|}. \end{array}$$
(10) Until min⁡ (‖**w**_*k*+1_ − **w**_*k*_‖, ‖**w**_*k*+1_ + **w**_*k*_‖) ≤ *ς*, otherwise go back to step (7).

## 5. Simulation and Discussion

To demonstrate the effectiveness of the proposed one-unit ICA-R algorithm, both artificial ECG data and real-word ECG data are used in the following two experiments. To quantitatively compare the performance of the proposed one-unit ICA-R and Mi's one-unit ICA-R [25], we adopt the individual performance index (IPI) as an indicator function, as was used in a previous study [11]. It is defined as

(40) $$\begin{array}{c} \text{IPI}=\left(\;\overset{M}{\underset{i=1}{\sum }}\frac{\left|p_{i}\right|}{\max_{j}\left|p_{j}\right|}\;\right)-1,\;j=1,\ldots ,M, \end{array}$$

where |·| denotes the absolute value operator, *p*_*i*_ denotes *i* element of the global system vector **p** = **w** ^*T*^ **B** **A** = (*p*_1_, *p*_2_,…, *p*_*M*_), **A** is mixing matrix, **B** is the whiten matrix, **w** is the separating vector of mixed signals after whitening, **w** ^*T*^ **B** is the global separating vector, *M* is the number of mixing signals, and max_*j*_ | *p*_*j*_| is to find the maximum valued among the elements in the vector **p** = (*p*_1_, *p*_2_,…, *p*_*M*_). The extracted signal by proposed method can be described as

(41) $$\begin{array}{c} y=w^{T}Bx=w^{T}BAs=ps=\overset{M}{\underset{i=1}{\sum }}p_{i}s_{i}. \end{array}$$

From (41), it can be easily seen that if **p** = (0,0,…, *p*_*k*_,…, 0), *p*_*k*_ ≠ 0, we will have the extracted signal *y* = *p*_*k*_ *s*_*k*_, which is just rescaled signals of *s*_*k*_. It is obvious from (40) that we have IPI ≥ 0 and that IPI equals zero if and only if **p** is a rescaled canonical base vector that only one element of vector is not zero. Therefore, the closer to a rescaled canonical base vector the global system vector **p**, the nearer to zero the IPI and thus the better the performance of one-unit ICA-R method.

In the experiments, we select *F*(*y*) = log cosh ⁡(*y*) as the nonquadratic function and *ε*(*y*, *r*) = *E*{(*y* − *r*)^2^} as the closeness measure function. Additionally, *g*(*y*) = *ε*(*y*, *r*) − *ξ*. We also use the corrected first derivative of *g*(*y*) as its first derivative. The corrected first derivative is given in [19] as

(42) $$\begin{array}{c} \nabla_{w}g\left(\;y\;\right)=E\left\{\;xg^{\prime }\left(\;y\;\right)\;\right\}=2E\left\{\;x\left(\;y-r\;\right)\;\right\}. \end{array}$$

In the following experiments, we set *ρ*, *η*, and *γ* all equal to one and perform 1000 trials for each data set.

### 5.1. Experiment with Artificial ECG Data

The artificial signals include the power line interference with 50 Hz, Gaussian noise, FECG, and MECG. The FECG and MECG are generated by using the ECG toolbox of Sameni [28]. The reference signals for the FECG and MECG are constructed with sign operation to roughly give the signs of the most data samples of the desired source signal [14, 15]. There are 5000 samples in the experiment. The mixing matrix is randomly generated as

(43) $$\begin{array}{c} A=\left[\begin{array}{cccc} 0.8925 & 0.0570 & 0.5044 & 0.9153\\ 0.0169 & 0.0590 & 0.4364 & 0.4911\\ 0.5165 & 0.4735 & 0.8193 & 0.7484\\ 0.0418 & 0.3840 & 0.4448 & 0.3421 \end{array}\right]. \end{array}$$

**Figure 1:**
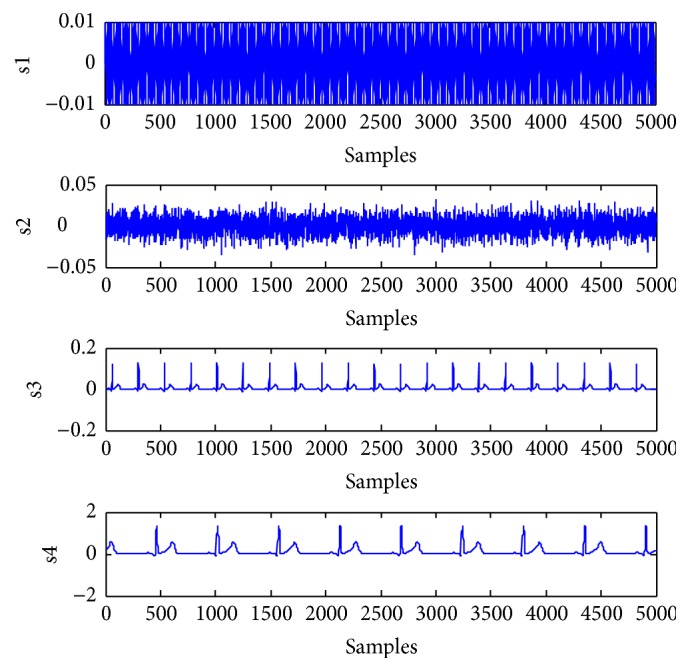
Four artificial source signals. (s1): power line interference. (s2): random Gaussian noise. (s3): FECG signal. (s4): MECG signal.

**Figure 2:**
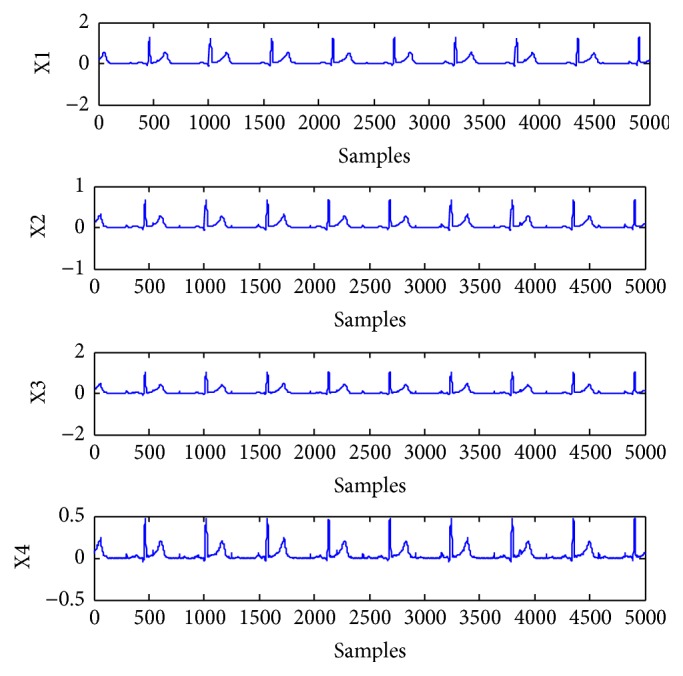
Mixed signals from sources in Figure 1.

**Figure 3:**
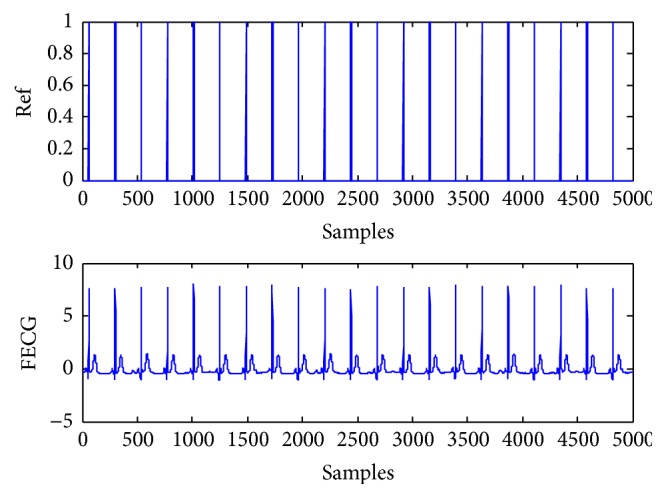
Reference signal and extracted artificial FECG signal. (Ref): reference signal for FECG signal. (FECG): extracted artificial FECG signal by the proposed method from mixed signals in Figure 2.

**Figure 4:**
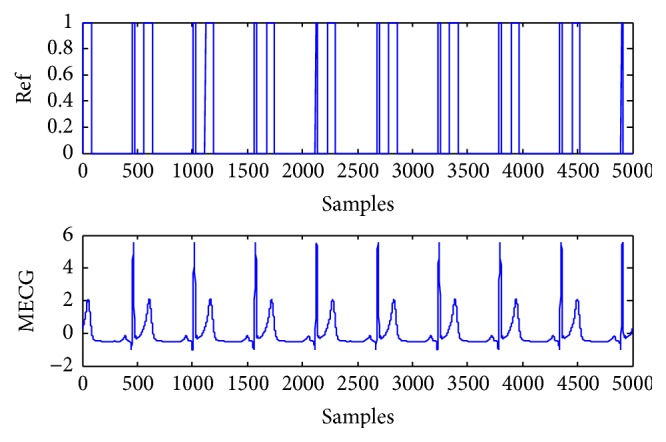
Reference signal and extracted artificial MECG signal. (Ref): reference signal for MECG signal. (MECG): extracted artificial MECG signal by the proposed method from mixed signals in Figure 2.

Four artificial source signals are depicted in Figure 1, which are considered independently of each other. s1 is the power line interference with sub-Gaussian distribution. s2 is the random Gaussian noise with Gaussian distribution. s3 and s4 are FECG and MECG with super-Gaussian distribution, respectively. The mixtures are shown as mixed signals in Figure 2. The FECG extracted by the proposed method and its reference are shown in Figure 3. The MECG extracted by the proposed method and its reference are shown in Figure 4. Comparisons of the extracted FECG and the extracted MECG are shown in Figures 3 and 4 with the desired signals s3 and s4 in Figure 1, respectively. It is evident that the waveforms of the extracted signals are most identical to the waveforms of the desired signals.

To quantitatively compare the performance of the proposed method and that of Mi's method [25], the IPI, defined in a previous report (40), are computed for the extraction results of both algorithms in the same experiment. Both algorithms use the same parameters and reference signal. The results are shown in Table 1, where it can be seen that the IPIs are the same for the proposed method and Mi's method. This means that both methods successfully extract the FECG and MECG with the same low IPI. However, the running time consumed by both methods is quite different. The running time of the method proposed here is roughly half of that of Mi's algorithm.

### 5.2. Experiment with Real-World ECG Data

**Figure 5:**
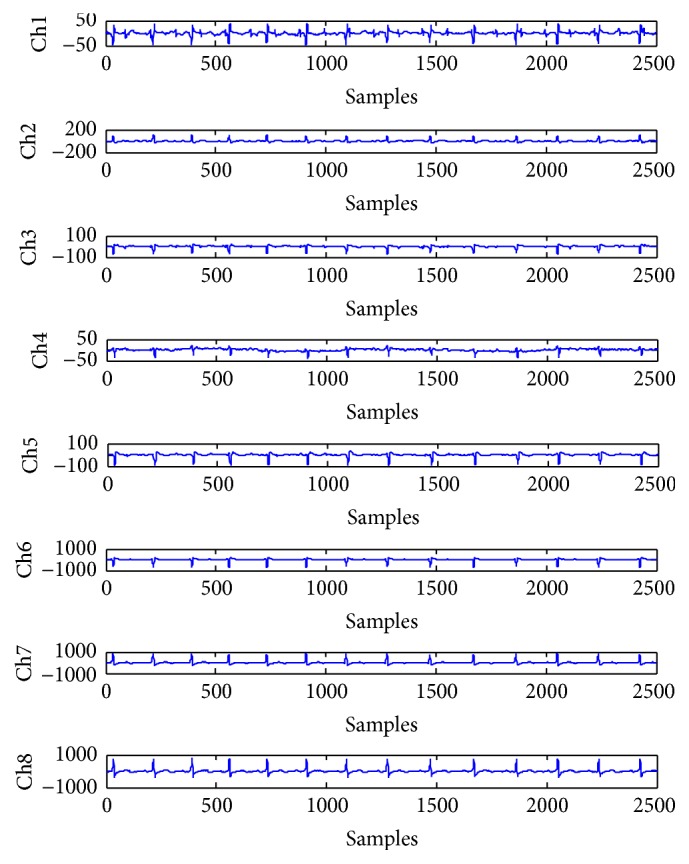
Real-world ECG recordings: the 8 channels of ECG recordings obtained from a pregnant woman with data length of 2500 samples and time of 10 s [27].

The real-world ECG data was contributed by De Moor [27] and has been widely used by other researchers [11–13, 25]. It was recorded over 10 s and sampled at 250 Hz by placing eight electrodes on different locations of a pregnant woman. The real-word ECG data are shown in Figure 5, where the signals Ch1–Ch5 were the recordings from five electrodes placed on the woman's abdomen. Accordingly, the FECG, respiratory motion artifacts, and the MECG were visible in these recordings. The signals Ch6–Ch8 were the recordings from three electrodes placed on the woman's thorax. In these thoracic measurements, the FECG was invisible because of the distance between the fetus and the chest leads.

In the Ch1 recording, the strong and slow heart belongs to the mother, while the weak and fast belongs to the fetus. Therefore, we can use the information about fetus in the Ch1 recording to construct reference signal for extracting the FECG. We use impulse series whose occurrence time is the same as that of the subpeaks in the first channel signal Ch1 as reference for extracting the FECG. The impulse series whose occurrence time is the same as the peaks of any channel signal were used as reference for extracting the MECG.

**Figure 6:**
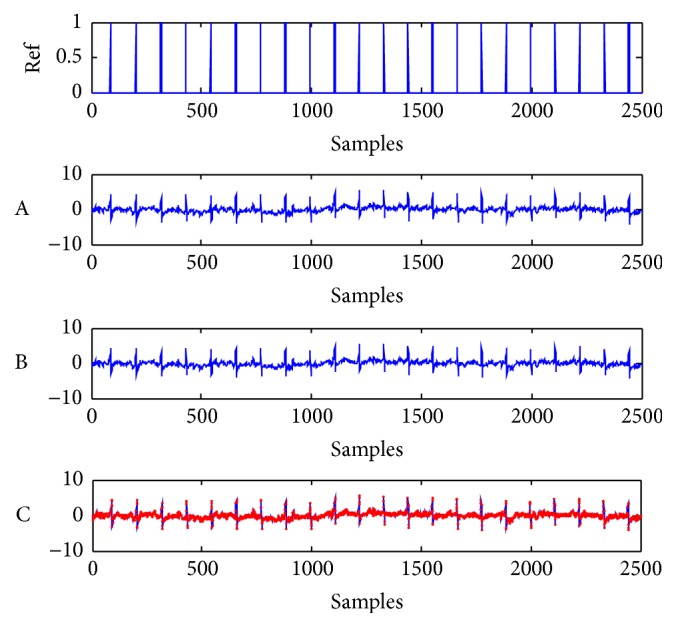
Reference signal and extracted FECG signals by both methods from the real-world ECG signals in Figure 5. Ref: reference signal for FECG signal. A-B: extracted artificial FECG signals by the proposed method and Mi's method, respectively. C: red and blue curves are exactly the same with the subfigures A and B, respectively.

**Figure 7:**
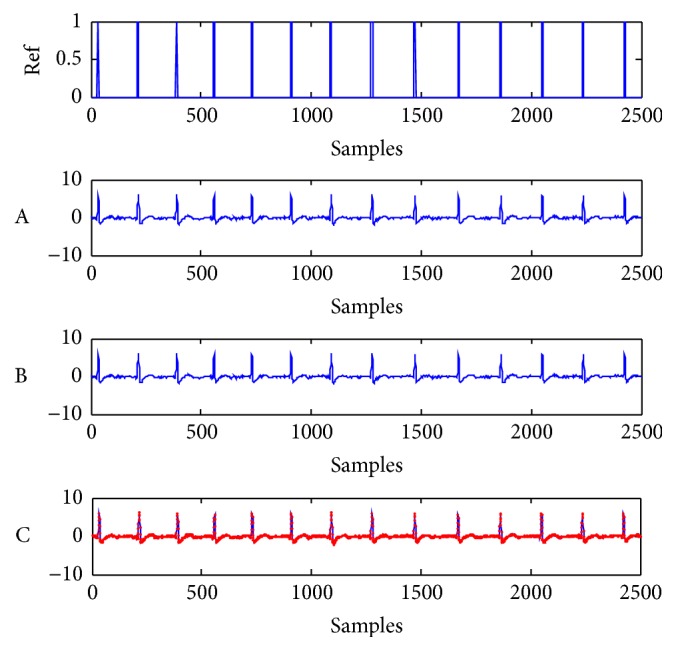
Reference signal and extracted MECG signals by both methods from the real-world ECG signals in Figure 5. Ref: reference signal for MECG signal. A-B: extracted artificial MECG signals by the proposed method and Mi's method, respectively. C: red and blue curves are exactly the same with the subfigures A and B, respectively.

The reference signal and the FECG signal extracted by both methods are shown in Figure 6, where the reference signal is denoted as Ref, the FECG extracted by the proposed method is denoted as A, the FECG extracted by Mi's method is denoted as B, and both extracted FECG signals are redescribed in C and represented with different color curves. The reference signal and the MECG signal extracted by both methods are shown in Figure 7, where the reference signal is also described as Ref, the MECG extracted by the proposed method is denoted as A, the MECG extracted by the previous method is denoted as B, and both extracted MECG signals are redescribed in C with different color curves. We can see in Figures 6 and 7 that the FECG and MECG are both successfully extracted by both methods. Since the mixing A and the pure FECG and MECG signals are not available for the real-word ECG recordings, the IPI performance cannot be computed. But we can compare the waveforms of extracted signals by both methods to estimate their relative error. Therefore, we redescribe the extracted FECG and MECG signal waveforms by both methods in the same subfigure C with different color curves in Figures 6 and 7, respectively. From the subfigure C in Figures 6 and 7, it can be seen that the different color curves are almost the same. This means that the proposed method and Mi's method have almost the same error. However, their consumed running time is considerably different, as shown in Table 2. The running time is approximately reduced to half of that of the Mi's algorithm.

## 6. Conclusion

In this paper a fast one-unit ICA-R algorithm for extracting ECG is proposed by simplifying the non-Gaussian measurement method of the extracted signal. In the proposed algorithm, the nonlinear function of the extracted signal is directly used as non-Gaussian measurement function. This avoids having to compute the difference between the function of the extracted signal and the function of the Gaussian random vector with the same mean and variance as the extracted signal. Centering and whitening are also used to preprocess the observed data to avoid computing the observed signal covariance matrix. As a result, the computation complexity for the one-unit ICA-R algorithm is greatly reduced.

The validity of the proposed algorithm is tested and compared to Mi's algorithm using artificial ECG data and real-world ECG data. Both experimental results demonstrate that the convergence rate of the proposed algorithm is two times faster than Mi's algorithm, while both methods have the same error.

## Figures and Tables

**Table 1:** Comparison of the IPI performance and time consumed by the proposed method and Mi's method.

Desired signal		FECG	MECG
Proposed	IPI	0.0144	0.0788
Mi [25]	IPI	0.0144	0.0788
Proposed	Time (s)	0.010359	0.014047
Mi [25]	Time (s)	0.025578	0.033813

**Table 2:** Comparison of the time consumed by the proposed method and Mi's method for the real-word ECG data.

Desired signal		FECG	MECG
Proposed	Time (s)	0.022828	0.022109
Mi [25]	Time (s)	0.050484	0.049234

## References

[1] Ferrara E. R., Widrow B. (1982). Fetal electrocardiogram enhancement by time-sequenced adaptive filtering. *IEEE Transactions on Biomedical Engineering*.

[2] Kanjilal P. P., Palit S., Saha G. (1997). Fetal ECG extraction from single-channel maternal ECG using singular value decomposition. *IEEE Transactions on Biomedical Engineering*.

[3] Khamene A. H., Negahdaripoure S. (2000). A new method for the extraction of fetal ECG from the composite abdominal signal. *IEEE Transactions on Biomedical Engineering*.

[4] de Lathauwer L., de Moor B., Vandewalle J. (2000). Fetal electrocardiogram extraction by blind source subspace separation. *IEEE Transactions on Biomedical Engineering*.

[5] Hasan M. A., Ibrahimy M. I., Reaz M. B. I. (2007). Techniques of FECG signal analysis: detection and processing for fetal monitoring. *WIT Transactions on Biomedicine and Health*.

[6] Sargolzaei S., Faez K., Sargolzaei A. Signal processing based for fetal electrocardiogram extraction.

[7] Sevim Y., Atasoy A. (2011). Performance evaluation of nonparametric ICA algorithm for fetal ECG extraction. *Turkish Journal of Electrical Engineering & Computer Sciences*.

[8] Liu G., Luan Y. (2015). An adaptive integrated algorithm for noninvasive fetal ECG separation and noise reduction based on ICA-EEMD-WS. *Medical & Biological Engineering & Computing*.

[9] Li Y., Nie W., Ye F., Li A. (2016). A fetal electrocardiogram signal extraction algorithm based on the temporal structure and the non-Gaussianity. *Computational and Mathematical Methods in Medicine*.

[10] Lee J., Park K. L., Lee K. J. (2005). Temporally constrained ICA-based foetal ECG separation. *Electronics Letters*.

[11] Lin Q.-H., Zheng Y.-R., Yin F.-L., Liang H., Calhoun V. D. (2007). A fast algorithm for one-unit ICA-R. *Information Sciences*.

[12] Zhang Z.-L. (2008). Morphologically constrained ICA for extracting weak temporally correlated signals. *Neurocomputing*.

[13] Yongjian Z. Constrain component extraction techniques.

[14] Li C., Liao G., Shen Y. (2010). An improved method for independent component analysis with reference. *Digital Signal Processing*.

[15] Lu W., Rajapakse J. C. ICA with reference.

[16] Lu W., Rajapakse J. C. (2005). Approach and applications of constrained ICA. *IEEE Transactions on Neural Networks*.

[17] Lu W., Rajapakse J. C. (2006). ICA with Reference. *Neurocomputing*.

[18] Hyvärinen A. (1999). Fast and robust fixed-point algorithms for independent component analysis. *IEEE Transactions on Neural Networks*.

[19] Huang D.-S., Mi J.-X. (2007). A new constrained independent component analysis method. *IEEE Transactions on Neural Networks*.

[20] Sun Z.-L., Shang L. (2010). An improved constrained ICA with reference based unmixing matrix initialization. *Neurocomputing*.

[21] Kavuri S. S., Zurada J. M., Rajapakse J. C. Evolutionary approach to ICA-R.

[22] Zhang J., Zhang Z. S., Cheng W. (2014). Kurtosis-based constrained independent component analysis and its application on source contribution quantitative estimation. *IEEE Transactions on Instrumentation and Measurement*.

[23] Wang Z. (2011). Fixed-point algorithms for constrained ICA and their applications in fMRI data analysis. *Magnetic Resonance Imaging*.

[24] Rodriguez P. A., Anderson M., Li X.-L., Adali T. (2014). General non-orthogonal constrained ICA. *IEEE Transactions on Signal Processing*.

[25] Mi J.-X. (2014). A novel algorithm for independent component analysis with reference and methods for its applications. *PLoS ONE*.

[26] Chen G. H., Qie L. F., Zhang A. J., Han J. (2016). Improved CICA algorithm used for single channel compound fault diagnosis of rolling bearings. *Chinese Journal of Mechanical Engineering*.

[27] De Moor D. Daisy: database for the identification of systems. http://www.esat.kuleuven.ac.be/sista/daisy.

[28] Sameni R. http://ee.sharif.edu/~ecg/Download/.

